# Data-Driven Framework for Understanding and Predicting Air Quality in Urban Areas

**DOI:** 10.3389/fdata.2022.822573

**Published:** 2022-03-25

**Authors:** Lakshmi Babu Saheer, Ajay Bhasy, Mahdi Maktabdar, Javad Zarrin

**Affiliations:** Faculty of Science and Engineering, Anglia Ruskin University, Cambridge, United Kingdom

**Keywords:** urban air quality, climate change mitigation, urban vegetation detection, regression based prediction algorithms, machine learning and deep learning algorithms, aerial view image recognition, cost effective modeling

## Abstract

Monitoring, predicting, and controlling the air quality in urban areas is one of the effective solutions for tackling the climate change problem. Leveraging the availability of big data in different domains like pollutant concentration, urban traffic, aerial imagery of terrains and vegetation, and weather conditions can aid in understanding the interactions between these factors and building a reliable air quality prediction model. This research proposes a novel cost-effective and efficient air quality modeling framework including all these factors employing state-of-the-art artificial intelligence techniques. The framework also includes a novel deep learning-based vegetation detection system using aerial images. The pilot study conducted in the UK city of Cambridge using the proposed framework investigates various predictive models ranging from statistical to machine learning and deep recurrent neural network models. This framework opens up possibilities of broadening air quality modeling and prediction to other domains like vegetation or green space planning or green traffic routing for sustainable urban cities. The research is mainly focused on extracting strong pieces of evidence which could be useful in proposing better policies around climate change.

## 1. Introduction

The quality of air that we breathe is an important factor for a healthy human life and is a major concern throughout the world in both, developed and developing countries. The ever-growing urban population and increased volume of industries and motorised vehicles in cities resulted in air pollution, affecting the environment and posing significant threats to human health. Maintaining clean air is essential for our well-being and sustaining life on our planet. To address these concerns, researchers have designed and developed several solutions for air quality analysis and evaluation. Early air quality evaluation methods relied on conventional statistical approaches and were restricted by limited accuracy and lack of flexibility (Kang et al., 2018). The advent of modern Artificial Intelligence (AI) techniques such as Artificial Neural Networks (ANN) opened up new possibilities for researchers around the world to find solutions to various problems affecting air quality and climate change (Rybarczyk and Zalakeviciute, 2018; Rolnick et al., 2019).

One of the domains that have gathered a lot of attention in recent years is air quality monitoring and urban city planning. Availability of Big data in domains like traffic management and air pollutants concentration monitoring systems can directly help us to plan our cities and traffic routes or even come up with policies and regulations to keep our carbon footprint under control. The main sources of air pollution in cities are emissions from different sources including traffic, industrial and household sources. But, in fact, there are other factors like vegetation and weather conditions that dictates the concentration of these pollutants in the air mainly through dispersion and absorption (Guan et al., 2016). To this end, it is important to understand the relationship of these factors with respect to pollutant concentration. Understanding these relationships can help in urban vegetation or traffic route planning to control this pollutant concentration.

One of the major factors affecting the air quality and concentration of pollutants in the atmosphere is the vegetation (Bealey et al., 2007). Impact of tree plantations in the urban area including highway borders have been investigated as an effort to improve urban air quality (Benjamin and Winer, 1998; Al-Dabbous and Kumar, 2014; Fares et al., 2016; Waters et al., 2021). Researchers have studied the influence of vegetation on both particulate and gaseous pollutants. Detailed reports have been generated by experts in the field to aid authorities in urban green space development (Bealey et al., 2007; Baldauf et al., 2013). There have been efforts in recent years toward sustainable urban transportation planning which in turn has also influenced the vegetation planted around the cities and highways (Baldauf et al., 2013).

Building on our initial studies (Babu Saheer et al., 2020; Babu Saheer and Shahawy, 2021), this research aims to generate a framework for monitoring and modeling the air quality for urban cities by understanding the different factors that influence the concentration of pollutants in the air. Integrating information from various sources including measured pollutant concentration, weather, traffic and other correlated features alongside understanding vegetation distribution around urban cities can help urban planners to build sustainable green spaces. Most of the aforementioned factors are available as public data through various monitoring services of the government or other non-profit organisations (Cambridge City Council, 2019; Highway England, 2019; Transport for London, London Air Quality, 2019). Also, the weather data that is usually monitored by meteorological societies is available as a live stream (Onal et al., 2017).

The vegetation itself may be a challenging factor to monitor. Some of the local authorities such as UK city councils have tried to maintain a record of tree plantations (London Local Authority Maintained Trees, 2019). But there are limited incomplete records of vegetation around the city. It would be easier to automatically detect this information from remote sensing or satellite images. Again, remote sensing using light detection and ranging (LIDAR) and drones would be expensive and not easy to scale. A cheaper and more convenient option to this end would be to use the Google Earth satellite images. According to their official product blog, “Google has collected 36 million square miles of high definition satellite images from various providers covering more than 98% of the entire population to see the world from above" (Lookingbill, 2019). These high quality aerial view images are aligned and stitched together using photogrammetry achieved through Machine Learning. Sources also mention that Google keeps updating these images on a regular basis. Based on this information, vegetation can be determined as tree crown recognition on these aerial view images. The main challenge with the satellite images is that there is no labeled data available to train tree crown detection models. Unsupervised or semi-supervised modeling techniques could be explored for detecting the vegetation from these images. To this end, the research presented in this paper first looks at detecting and understanding vegetation as number and species of trees in and around an urban area from Google Earth satellite images using different deep learning models. Once both the vegetation and weather data is aligned and collated, the framework can be put together to understand how the air quality in terms of pollutant concentration is being affected by these factors. Further, such a framework can be extended to predict the air quality in other regions and even suggest city planning in terms of roadside vegetation or local green spaces.

The main aim of this research is to come up with a sustainable and affordable framework for air quality modeling by integrating pollutant concentrations with the weather conditions and vegetation information. This scalable framework can be easily adapted to work for any international location. A prototype implementation of this framework is validated in this paper for the city of Cambridge. City of Cambridge has been chosen for this pilot study as it has other factors mentioned in the framework publicly available being monitored by local authorities. This paper makes several contributions to the air quality research as listed below.

1. The main novelty of the paper is the proposed framework which aims to bring together different aspects related to air quality including weather, vegetation, and other factors to predict air quality in any desired location of the world. There have been other studies that looked into the influence of one factor or the other separately on air quality from historic data. Detailed comparison of other state-of-the-art research is presented in Section 2. But, this research proposes a framework to collect all the information simultaneously in a cost-effective sustainable manner and combine them together in a model to predict the desired pollutant concentration of a region.
2. Novelty of the framework includes the innovative approach to detect the vegetation using self-supervised deep learning models on aerial view images and incorporated this into the framework.
3. Apart from the framework, the research novelty includes a case study implementing the framework on Cambridge city and testing with multiple machine learning algorithms compared with traditional mathematical modeling and advanced deep learning techniques.
4. The novelty includes new features engineered to improve the performance of the air quality models including information on seasonal trends, traffic trends (weekend/weekday/working hours), and vegetation information within multiple radii (Number of trees within 100 m, 250 m, 500 m).
5. Multiple modeling techniques were investigated including statistical (ARIMA), linear models (linear regression, support vector regression (SVR), and polynomial regression), non-linear (SVR with polynomial and radial basis function kernels and its combination) and deep learning (Long Short Term memory) models.

As the next step of this research, the team is building a portable cost-effective custom air quality (including different pollutants like PM, *NO*_*x*_, *SO*_2_, O3, *CO*_2_, etc.) and weather (esp. temp, humidity, and wind conditions) monitoring devices to replace the industrial grade sensor data used in this study. This will drastically expand locations of the air quality monitoring/predictions (for e.g., every street rather than just around the four government established monitoring stations in Cambridge) and pave way for micro-climate modeling. This framework should essentially benefit under developed countries struggling to afford the industrial grade sensor. Novelty of the work includes determining the vegetation from satellite images (could be replaced with drone imagery in the future) to be incorporated into an air quality framework. This is just a first step toward a long term research which will look into refining the aspects of the framework and modeling including the vegetation factors (tree species, difference in vegetation during seasons, etc.), and other climatic aspects of soil absorption factors, atmospheric stratification, solar radiation effects.

The rest of this paper is organized as follows. Section 2 discusses the details of earlier work in this domain. The details of the data set followed by data analysis and pre-processing is presented in Section 3. The novel methodology for estimation of the vegetation from aerial imagery is presented in Section 4. Section 5 discusses different approaches undertaken in this research along with the results and discussions in Section 6.

## 2. Related Work

The increasing concentration of greenhouse gas emissions is considered as the prime cause of climate change and air quality degradation over the last three decades and many studies focused on the way in which this can be monitored and mitigated. Air quality, and in specific, the impact of vegetation on air quality has been in the spotlight of many researchers for the last decade. Studies show vegetation and trees can both influence the atmospheric composition of trace gases and enable dispersion and deposition of air pollutants, thus affecting the concentrations of pollutants that populations in urban areas are exposed to. However, the research outcomes are variable and none of these studies show any definite outcome on this matter.

There have been recent studies modeling urban Air quality (Liang and Gong, 2020; Wolf et al., 2020), most of which do not consider other related factors. A study by Duarte et al. (2015) investigates the impact of vegetation on urban micro-climate and the warming effect resulting from an increase in built density in a subtropical climate. They have measured air temperature, relative humidity, solar radiation, soil temperature, wind direction, and speed in Bela Vista district of São Paulo, Brazil to pre-calibrate ENVI-met V4 preview prior to parametric simulations. Also, they have set up a Campbell Scientific meteorological station in the center of the central and densest block to monitor the micro-climate effect. The diurnal variation of air temperature and relative humidity have been measured and monitored on an hourly basis. They have measured the effect of vegetation on micro-climates by considering the tree's shadowing and physiological process of evapotranspiration. This study showed that the presence of vegetation can significantly reduce the surface temperature and mean radiant temperature of the urban area.

In another study, Holnicki and Nahorski (2015) showed how emission uncertainty of air pollutants generated by the industry, traffic, and the municipal sector relates to concentrations measured at receptor points in the Warsaw metropolitan area of Poland. This study identified the transportation system as the main source of adverse environmental impact. Several types of urban atmospheric pollutants including PM_10_, PM_2.5_, NO_*x*_, SO_2_, and Pb were included in this study and analyzed using the *Monte Carlo* technique to identify the key uncertainty factors. Zhu et al. (2018) attempted to tackle air quality forecasting by predicting the hourly concentration of air pollutants such as Ozone, PM_2.5_, and SO_2_ on the basis of meteorological data of previous days by formulating the prediction over 24 h as a multi-task learning (MTL) problem. This study also proposed a consecutive hour-related regularization to achieve better performance figures.

A study by Kleine Deters et al. (2017) offers a machine learning model based on Boosted Trees and Linear Support Vector Machines to analyse meteorological and pollution data collected from the city of Quito, Ecuador to predicting the concentrations of PM_2.5_ from wind speed and direction and precipitation levels. This study shows aforementioned machine learning models are capable of accurately predict concentrations of PM_2.5_ from meteorological data. Another study by Zalakeviciute et al. (2018) investigates the impact of meteorological and topological conditions on urban air pollution using data collected from the city of Quito, Ecuador. This study specifically investigates the impact of the relative humidity (RH) on the daily average PM_2.5_ concentrations. Results of this study show a positive correlation between daily average urban PM_2.5_ concentrations and the RH in traffic-busy central areas, and a negative correlation in the industrial city outskirts.

Zhang et al. (2019) aimed for tackling issues such as the instability of data sources and the variation of pollutant concentration along time series based for a better air quality predictive model. This study measured PM_2.5_ concentration in over 35 air quality monitoring stations in Beijing and used the LightGBM model and forecasting data to address the issue of high-dimensionality. Ameer et al. (2019) proposed a comparative analysis of four regression machine learning techniques including decision trees, random forest, gradient boosting, and multi-layer perceptron for predicting air quality in specific PM_2.5_ atmospheric pollution in smart cities. This study shows that the Random Forest regression model was the best technique for pollution prediction in urban environments. A similar study by Aditya et al. (2018) attempted to predict air quality and PM_2.5_ atmospheric pollution using logistic regression. A comprehensive exploratory study by Rybarczyk and Zalakeviciute (2018) attempted to investigate the efficiency and performance of various machine learning techniques for outdoor air quality and atmospheric pollution modeling.

Rao et al. (2019) proposed an efficient approach for modeling and prediction of air quality using long short term memory (LSTM) Recurrent Neural Networks. This study attempt to capture the dependencies in various pollutants such as PM_2.5_, PM_10_, SO_2_, NO_2_, and Ozone to perform air quality prediction. RNN-LSTM allows modeling of temporal sequence data of each pollutant for forecasting hourly-based concentrations. Similarly, Belavadi et al. (2020) proposed an air quality forecasting architecture that gathers real-time air pollutant concentration including SO_*x*_, PM_2.5_, CO, and LPG using Wireless Sensor Networks (WSN) and real-time air quality data API and then uses LSTM-RNN to forecast future air pollutant concentrations. Masmoudi et al. (2020) attempted to predict multiple air pollutants concentrations including NO_*x*_, Ozone, and SO_2_*via* a novel feature ranking method that is based on a combination of Ensemble of Regressor Chains and the Random Forest permutation importance measure. Feature selection allowed the model to obtain the best subset of features. Harishkumar et al. (2020) proposed an air pollution forecasting model for PM_2.5_ atmospheric pollution using a machine learning regression model.

There are other studies that look at the satellite images to estimate the pollutants directly from images (Fang et al., 2016; Chen et al., 2018; Sun et al., 2019; Kalajdjieski et al., 2020; Shin et al., 2020). All these studies work for only particulate matter and not for gaseous pollutants. Our proposed research looks at both gaseous and particulate matter and uses the satellite imagery for vegetation detection not for pollutant detection. The pollutants in our proposed framework will be monitored through reliable sensors. Inclusion of weather parameters in air quality modeling has shown promising results (Kalajdjieski et al., 2020; Gonzlez-Enrique et al., 2021). Deep Learning models mainly LSTM based RNNs are being popularly used for both univariate and multivariate (with exogenous features) time series pollutant data. Different configurations of LSTM mainly cross-validation procedure for time series (LSTM-CVT) were compared with basic (Artificial neural networks) ANNs by Gonzlez-Enrique et al. (2021) for *NO*_2_ in the Bay of Algeciras (Spain). It was found that exogenous variables like weather parameters have shown considerable improvement in performance. LSTMs have also been used in traffic forecasting (Awan et al., 2020) and pollution classification (Arsov et al., 2021). Our research compares different machine learning models ranging from linear regression to multiple kernel based SVR techniques with both traditional mathematical models like ARIMA and the popular LSTM based deep learning models. Also, this work proposes to use more factors like vegetation and seasonal information on top of the previously suggested weather-based exogenous features.

A study by Tallis et al. (2011) proposed a predictive model to understand the role of urban trees in removing PM_10_ from urban air in Greater London. The research identified that the planned 10% increase in tree area within Greater London (from the current 20–30%) by 2,050 increases the annual PM_10_ removal from the current range of 852-2121 tonnes (0.7–1.4%) to 1,109–2,379 tonnes (1.1–2.6%). It was also identified that the increased deposition would be greatest if a larger proportion of coniferous to broad-leaved trees were used around the polluted areas. This study proposed two different approaches in order to determine the relationships between the amount and type of tree cover and PM_10_ uptake. The first approach measured PM_10_ downward flux relative to the urban tree canopy using deposition velocity and pollutant concentration while the second approach used species specific deposition velocities to estimate the PM_10_ uptake. The main drawback of this study is the lack of in-site validation. Issues like the sensitivity of selected species to atmospheric pollution and climate change, aesthetic appeal, biodiversity, soil factors, maintenance costs, and the land availability for planting programs have also not been considered.

In another study, Yang et al. (2015) investigated the suitability of common urban tree species for controlling PM_2.5_ pollution. This study developed a ranking approach to evaluate the PM_2.5_ removal efficiency, impacts on air quality, and the adaptability to urban environments of commonly occurring urban tree species. It was suggested to use species with high PM_2.5_ removal efficiency in urban greening projects. However, in the real world, PM removal efficiency is not the most important criterion for urban planting. The ability of the species to adapt to urban abiotic and biotic stresses such as compacted soil, water-logging, droughts, pests and diseases, and air pollutants are the most important factors in urban planting programs. The results of this study showed that the most frequently occurring urban tree species were not the best performers in removing PM_2.5_. Among the ten most frequently occurring tree species in the dataset, only three species namely, London plane, Silver maple, and Honey locust were ranked above average in capturing particulate matter. This study suggests conifer species have high PM_2.5_ removal efficiency while it is robust to urban abiotic and biotic stresses. A study by Yang et al. (2005) looked into the impact of the urban forest on air pollution in the city of Beijing. They relied on satellite image analyses and field surveys to establish the characteristics of the current urban forest in the central part of Beijing. Satellite images were obtained from EROS Data Center and captured by Landsat's Enhanced Thematic Mapper covers the Beijing region. This study attempted to create a model to quantify the major air pollutants including SO_2_, NO_2_, CO_2_, PM_10_, and O_3_ that are reduced from the atmosphere by urban forest in the central part of Beijing. This study also investigated the Biogenic Volatile Organic Compound (BVOC) emission sourced from the urban forest. The results of this study showed that 2.4 million trees in Beijing central reduced over 772 tons of PM_10_ and over 0.2 million tons of CO_2_ stored as biomass in a year.

Wilkes et al. (2018) used multi-scale LiDAR imaging including terrestrial and airborne laser scanning to estimate urban ground biomass for the London Borough of Camden, UK. An airborne laser scanning was used in the first instance to create clusters of feature sets that represented a wide range of tree structures typical in an urban setting. Then, terrestrial LiDAR measurements were used to derive allometry that uses structure metrics to identify individual trees and subsequently estimate the above ground biomass. This study used two relatively expensive imaging techniques including terrestrial and airborne laser scanning to estimate the above ground biomass which is less preferable in the real world. A similar study by Li et al. (2020) attempted to estimate urban vegetation biomass in the east Chinese city of Xuzhou using a combination of field observations and Sentinel satellite images. Field measurements were used to identify the Quadrat biomass using the allometric biomass equations. Vegetation biomass models were constructed using remote sensing Sentinel satellite images. This study attempted to identify the capability of Sentinel-2A data to estimate urban vegetation biomass and examine whether vegetation type-specific modeling can improve estimation accuracy. Similar to the earlier study, this approach is also less preferable in the real world as it requires labor-intensive and expensive field observations and manual surveying. Similarly, studies including Reitberger et al. (2009), Lahivaara et al. (2013), Zhang et al. (2014), and Qin et al. (2014) used airborne LiDAR or a combination of airborne and point clouds LiDAR technologies for individual tree crown detection. There are other types of studies like (Kraft et al., 2019) who aimed to model vegetation dynamics in conjunction with climate change impacts. Kraft et al. (2019) used LSTM network and multivariate predictors to model earth system variables to create a global model for vegetation dynamic state. The authors have used 33 years of climate variables in addition to static soil and land cover characteristics to model daily satellite-based observations. The proposed LSTM based model was able to learn the dynamicity of vegetation through temporal and global spatial variables. However, the focus of the study is not on air quality.

With an aim to promote urban tree management, Branson et al. (2018) created up-to-date catalogs of urban tree population using publicly available TreeMapLA Los Angeles tree inventory along with aerial and street view images of Google Maps. This study also aimed to create a change-tracker model that recognizes changes of individual trees at city-scale, which is essential to keep an urban tree inventory up-to-date. The study first scraped available aerial images and street view panoramas of the city of Pasadena from Google Maps. Then, a tree detector and a tree species classifier were separately trained using labels from the TreeMapLA dataset. The trained tree detector predicted all unseen available tree images and then projected them from the image space to true geographic positions. Larsen et al. (2011) conducted a comparison study of six individual tree crown detection algorithms and evaluated their performance using an image dataset containing six diverse forest types at different geographical locations in three European countries. This study showed that the majority of algorithms were straggling with individual tree crown detection in non-homogeneous images of forestry. More related literature on this topic is summarized in Section 4 which presents our approach of self-supervised tree crown detection from Google Earth images. Some of the limitations in the earlier attempts of vegetation or tree crown detection in urban areas and mapping this information to an air quality modeling framework have been discussed above. Furthermore, none of these aforementioned projects consider the factors of weather and climatic conditions or other factors for a generic air quality modeling framework. As mentioned earlier, our research proposes a comprehensive and affordable framework for urban air quality modeling.

## 3. Data Mining and Processing

In order to build a prototype for the aforementioned framework, the first step is to acquire different datasets for the selected region (Cambridge). Three vital features are required to build this framework: the number of trees, the pollutant concentration, and the weather data, all of which belong to the bounded geographical region and with in the same time period. Collecting these datasets is not a trivial task, especially since the data needs to be from exactly the same time period and location. Cambridge city council monitors pollutant concentrations that are published online and weather data can be acquired from the local weather station. Deep Learning based techniques on aerial view images had to be developed in order to properly infer the vegetation data as tree locations to estimate the count of trees around the points where the pollutant concentrations are monitored. The following sub-sections provide the details of the different datasets used in this framework.

### 3.1. Pollutant Concentration Data

The pollutants monitored for air quality can be categorized into two classes–gaseous (*CO*_2_, *SO*_2_, *NO*_*x*_, and *NO*_2_) and particulate matter (*PM*_10_, *PM*_2.5_). It could be postulated that the trees help absorb only gaseous pollutants. But, there have been reports on certain types or species of trees that could help absorb the particulate matter as well (Bealey et al., 2007; Chen et al., 2017). Our initial study (Babu Saheer et al., 2020) performed data analysis separately on the effects of vegetation on the gaseous and particulate matter for the London city. Even without looking at the tree species information, the results were positive as to the effect of trees with a strong negative correlation to pollutants. The particulate matter may seem to have more effect on general health rather than climate change. But, it is known that the particulate matter has fractions of elementary carbon (Chernyshev et al., 2019) which results in global warming and hence affecting climate change directly. These types of pollutants also need to be included in such studies.

The current study looks at the pollutant information collected by Cambridge council for the Cambridge City. The data is available *via* the Air Quality England website. There are monitors at four different locations in the city. These are Cambridge Gonville Place, Cambridge Montague Road, Cambridge Newmarket Road, and Cambridge Parker Street. Each of these locations records both gaseous and particulate matters at regular intervals (refer to Table 1). This time series data is available for every hour and could be easily aggregated for different time intervals if required. As shown in the table, not all locations are monitoring the same pollutants. This would make it difficult to build combined models using the parameters from different locations. In order to keep the prototype of this modeling framework simple, we currently focus on only a particulate matter (*PM*_10_) and a gaseous pollutant (*NO*_2_). Other pollutant types can be easily plugged into the framework.

**Table 1:** Pollutants monitored in Cambridge city.

**Sensor location**	**PM10**	**PM_**2.5**_**	**NO**	**NO_**2**_**	**NO_***x***_**
Gonville place	✓	✓	✓	✓	✓
Montague road	✓	X	✓	✓	✓
Newmarket road	X	✓	✓	✓	✓
Parker street	✓	X	✓	✓	✓

Ideally, it might be postulated that the emissions information also needs to be included in the analysis and modeling of air quality, rather than just the pollutant concentration. Pollutant concentration refers to the measured value of the pollutants in the air monitored in μ*g*/*m*^3^ as frequently as every hour or half hour while the emissions are estimated from the various sources in terms of tons/year over a larger period such as a year. Depending on the types and number of sources at a location, the accuracy of this estimation might widely vary. Usually, transport is deemed as the main source of emissions, but road transportation constitutes roughly 25 to 35% of the total emissions in the Greater London region (London Atmospheric Emissions Inventory, 2016). The concentrations of pollutants are measured regardless of the source. Another limitation with the current emissions and pollutant concentration datasets is that the type of pollutants monitored may not be the same. For instance, the London dataset studied earlier, had the emissions data with *CO*_2_, *NO*_*x*_, *PM*_2.5_, and *PM*_10_ and the concentration data with *NO*_*x*_, *NO*_2_, *PM*_2.5_, and *PM*_10_. There were only *NO*_*x*_, *PM*_2.5_, and *PM*_10_ that are aligned and could be studied in parallel. Our earlier research, focused only on these 3 pollutants to study the direct relations between emissions and concentration. There are no emissions data included in the current study, as our focus was to have more accurate modeling and this could be added as a future improvement to this research.

### 3.2. Weather Data

The weather data is available from meteorological stations. Several studies have shown that the weather information in terms of wind speed, direction, humidity, temperature, dew point temperature, atmospheric pressure, rain and sun hours are important factors in determining the concentration of the pollutants in the air (Jhun et al., 2015). The direction and speed of wind can effect the concentration of PM particles. A study observed that the presence of wind in east, south, and south-east directions can increase the concentration of PM_2.5_ particles in the UK. A 25–50% of this increase is attributed to the PM_2.5_ carried over to the UK from continental Europe. Low wind speed also increases the PM_2.5_ concentration (Graham et al., 2020). Our research looks at the weather information from the weather stations in Cambridge. This results in only a single reading for all locations within Cambridge at each point in time. Ideally, the micro-climate modeling at each location could help in more accurate modeling. Again, this is left as a future study. All the locations in Cambridge are close by and it is reasonable to consider the reading from the single weather station for the scope of this study. The weather features used in our research are shown in Table 2.

**Table 2:** Weather data variables and their units in the data set.

**Weather data variable**	**Unit**
Temperature	Degree celsius (°C)
Dew point temperature	Degree celsius (°C)
Pressure	Millibar (mBar)
Wind speed	Knots (kts)
Wind direction	Direction (South-East, East etc)
Sunshine hours	Hours (hrs)
Rain	Millimeter (mm)
Maximum wind speed	Knots (kts)

### 3.3. Vegetation Data

The vegetation itself may be a tricky factor to measure. Some of the local authorities such as UK city councils have tried to maintain a record of tree plantations (London Local Authority Maintained Trees, 2019). But these are often limited and incomplete records of vegetation around the city. It would be easier to automatically detect this information from remote sensing or satellite images. Remote sensing using LIDAR and drones would be expensive and not easy to scale.

In order to build a scalable system capable of having global applications, an easily accessible source of data is required. Estimating the number of trees manually is not feasible. This specific obstacle led to the development of one of the cornerstones of the framework: the estimation of tree data through aerial images (Babu Saheer and Shahawy, 2021). Aerial images can be captured by iteratively looping over a bounded geographical region with Google Maps API using a sliding window. These aerial images of the region can be automatically analyzed to gather the vegetation details as tree counts. In this study, the regions explored were the Camden borough in London and the entire Cambridge city (CB1 to CB25). The Section 4 discusses in detail how the vegetation can be estimated as tree counts through tree crown detection on Aerial Google Earth images.

## 4. Mining the Tree Data

As mentioned earlier, leveraging the deep learning based image recognition on aerial view images could provide a good estimate of the vegetation information. In the absence of methods to capture these images in a cost-effective fashion, the research explored the use of Google Earth aerial view images as the source of data for a specific geographical location. Multiple self-supervised or semi-supervised or even unsupervised training techniques are needed to be experimented for this task in the absence of labels on the Google Earth images. Tree crown detection or delineation has been a popular domain of research to estimate the crown of the trees from aerial view remote sensing images. The technique could detect the tree counts as well as species or health of the trees. The proposed methodology in this research is referred to as “tree recognition” or “tree crown recognition.” The methodology estimates the bounding boxes on tree crowns in these images and thus helps to count the number of trees based on these tree crowns for each RGB image. The current research is extending this method to species detection as well (Waters et al., 2021).

Remote sensing is a very popular domain of research. The traditional mathematical modeling like canopy height model and image segmentation has been investigated by Wu et al. (2019). The popular techniques like point cloud detection (PCS), watershed, polynomial fitting, and individual tree crown segmentation (ITCS) were investigated and resulted in comparable scores. Deep learning methodology has gained momentum and is recognized as the most popular technique for any image recognition task including vegetation prediction (Guirado et al., 2017; Ayrey and Hayes, 2018) or scene classification from high-resolution multi-band images (Zhao et al., 2017) or RGB images (Hu et al., 2015). Different topologies of Convolutional Neural Networks (CNNs) based on pre-trained models like ResNet have shown significant improvement in precision (12%) and recall (36%) for (Guirado et al., 2017). The labor behind manual labeling has been the main concern with the use of supervised training algorithms. Labeling around 50,000 images of coffee scenes data (Hu et al., 2015 or 2,100 images of UC-Merced dataset (Castelluccio et al., 2015) is not scalable. Hence, unsupervised or semi/self supervised algorithms are desired (Wallace et al., 2014; Weinstein et al., 2019). The semi-supervised approach makes hand corrections on an already estimated initial labels with minimal training on high density LiDAR tree images (Wallace et al., 2014; Weinstein et al., 2019).

Most of the aforementioned work was modeled on forest or wooded areas with thickly populated trees of the same species. Aerial images are mostly from high resolution multi-spectral view data. The urban vegetation detection is more challenging due to the fact that the trees are from diverse species and scattered sparse distribution around the city. There is no labeled data available to build these models and the available data is a low resolution RGB images. As mentioned earlier a self-supervised approach was proposed by Weinstein et al. (2019) used LiDAR data to initialize the models and iterated on hand corrected noisy labels to refine the models. The research hand labeled 2,000 images which is claimed to be a decent set to get a reasonable performance from the model. The final model achieved recall of 0.69 and precision of 0.61. Labeling 2,000 images still may not be a feasible option. The vegetation detection is part of the aforementioned framework of pollutant monitoring and may not need very accurate counts. The work looks at small radii around the established pollutant monitors. Our earlier work (Babu Saheer et al., 2020) on London pollutant study based on noisy list (not very accurate count) of tree dataset from council provides further confidence on the relative influence of vegetation data for this framework. Our proposed approach is inspired by the earlier work of Weinstein et al. (2019) and expands it to a fully self-supervised approach without any hand labeling. This research investigates different approaches to estimate the vegetation from aerial view Google Earth images. Existing urban tree detection resources like Pasadena urban trees dataset and model (RegisTree Wegner et al., 2016) or tree crown recognition model named DeepForest (Weinstein et al., 2020) could be explored for building this self-supervised model. Number of experiments were performed using these two resources to identify a tree crown recognition model without the tedious effort of hand-labeling large amounts of aerial images.

Wegner et al. (2016) came up with a model to catalog public objects using both street and aerial view images in selected cities. A comprehensive dataset called the Pasadena dataset includes more than 80,000 trees labeled with species and corresponding geographic location on more than 100,000 multi-view Google images (map, street and aerial images). The multi-view modeling considerably improves the performance to 71% compared to the 42% detection rate achieved by the single view processing. The dataset only contains names of trees or species associated with each image and does not localize the location of the trees which is what is required in this work. Tree crown data could be synthesized and led to emergence of DeepForest model (Weinstein et al., 2020). DeepForest is an open-source Python package released with one pre-build model trained on data from the National Ecological Observatory Network (NEON) using a semi-supervised approach from Weinstein et al. (2019). It might be feasible to leverage this model to train a tree detection model using Google Earth images of Cambridge city. Millions of synthesised tree crown images were used to pre-train the model and may be identified as the baseline for tree detection similar to VGGNet or InceptionNet for image recognition. Pre-trained models can be used for building new models using the transfer learning technique (Shin et al., 2016). The technique leverages on the existing model using it as either a starting model to tune the parameters or using the model as a feature extractor to feed into a new model adapted to the new task. Given huge efforts in labeling remote sensing data, transfer learning is a very commonly used technique (Bonet et al., 2020). Identifying DeepForest and transfer learning as a possible approach for this task, the next step is to collect all the available data.

### 4.1. Data Mining and Pre-processing

**Figure 1:**
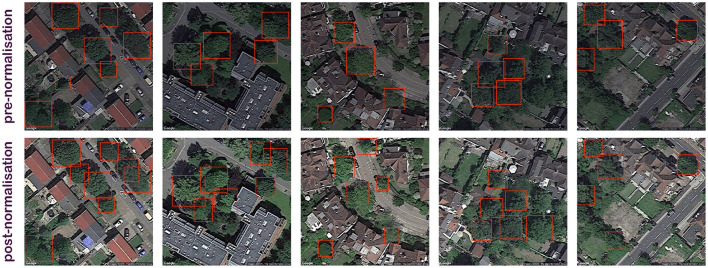
The impact of brightness, saturation, and normalization processes on tree detection performance. Normalized images are exhibiting a noticeably better detection accuracy.

As mentioned earlier, Google earth images were downloaded using Google maps API. The images supplied by Google are a combination of Satellite and Aerial view (Drone-style or airplane) images of RGB quality (as per sources). The set has mixed resolution on the images but is good enough to visually identify trees. The images were downloaded and followed through a pipeline of data pre-processing, filtering, and finally tree detection modeling. Square images at zoom level of 20 were downloaded using the Google map API covering a bounded geographical region of 70 m^2^. A sliding window of 70 m was used to download non-overlapping images. The anchoring point for each image was the top left corner represented by a pair of latitude and longitude values. Offsets were applied on these coordinates to download the image of the next adjacent geographical location. In order to make this calculation, one could assume that the point that is *r* meters away at a bearing of θ degrees east of north is displaced by r*cos(θ) in the north direction and r*sin(θ) in the east direction. Now given that the Earth's ellipsoidal curved shape needs to be accommodated rather than considering it as a plane surface, the longitude offset needs to be a function of the latitude offset. The aforementioned process was applied to downloaded aerial view images for two different locations—Cambridge and Camden (Borough in London) resulting in more than 500,000 images. Samples of these images are illustrated in Figure 1. The images represent urban scenery which unlike forest or wooded regions has multiple objects rather than just trees which are sparsely distributed and hard to differentiate from bushes.

The downloaded Google images could be further normalized to improve their quality similar to the images in the Registree dataset. The image properties mainly saturation, brightness and contrast could be separately normalized. The perceived values for these image properties are calculated from the dataset. All the images are normalized as follows to obtain unified image quality using pre-defined threshold (*P*_*bϕ*_, *P*_*sϕ*_, *P*_*cϕ*_) along with perceived stats (*P*_*bμ*_, *P*_*sμ*_, *P*_*cμ*_).


$$\begin{array}{l} \left(P_{b\phi }-P_{b\mu }\right)/P_{b\phi }\\ \left(P_{s\phi }-P_{s\mu }\right)/P_{s\phi }\\ \left(P_{c\phi }-P_{c\mu }\right)/P_{c\phi } \end{array}$$

Here b, s, and c represent the three image properties brightness, saturation, and contrast, respectively. Figure 1 represents some sample images with the aforementioned normalization. It can be seen that the trees are more visible in these normalized images.

### 4.2. Tree Detection Models

The pre-processed dataset can be now used for building a tree crown recognition model. As mentioned earlier, several approaches (including YOLOv3 based generic object recognition system) were tested and only the best performing model is presented in this paper. Due to the lack of labels in the dataset, a pre-trained model is required which could be minimally tuned to fit the task. The DeepForest model as discussed earlier is able to predict the bounding boxes of tree crowns on input images. The model itself was trained using the semi-supervised approach with synthesized images which are further optimised by retraining with hand-labeled data.

DeepForest model could be directly used to recognise the tree locations on the aerial view data downloaded as RGB images. The DeepForest architecture represents a similar RGB normalization block as mentioned previously (Section 4.1) for data pre-processing and image enhancement. The downloaded images when normalized had considerable improvement in performance without any retraining or tuning of the DeepForest model. It can be observed in Figure 1 that the number of bounding boxes for identified trees have drastically increased for the normalized images. But the overall performance can still be improved with self-supervised training. The initial performance of the model on unseen data downloaded from the Camden Borough in London has an average confidence score of 31.2%.

In the absence of good quality labeled data, transfer learning has emerged as one of the popular techniques to adopt (Shin et al., 2016). There may be data or tasks very close or similar to the one being addressed. These models or data can be leveraged to build a base model which can be further tuned or adapted with the limited data at hand. Transfer learning method has been very popularly used in image recognition tasks where there are big datasets like ImageNet rendering exceptionally powerful models like ResNet, InceptionNet, VGG16, or VGG32 etc. The convolutional layers in these popular CNN models could act as feature extractors to extract image specific features which are then used to train the feedforward fully-connected or softmax layers of the model. The same fine tuning can be tried on the DeepForest which addresses a similar task as this research, the tree crown recognition. Even for fine-tuning the model, there needs to be some or minimal amount of labeled data. In the absence of this, an approach similar to Weinstein et al. (2019), self-supervised learning is being used. But, the main difference for the proposed novel approach is unlike (Weinstein et al., 2019), this research will try to completely automate the re-training or tuning process by avoiding any hand corrections of the labels. Hence, there is no human effort or intervention involved in the whole process.

**Figure 2:**
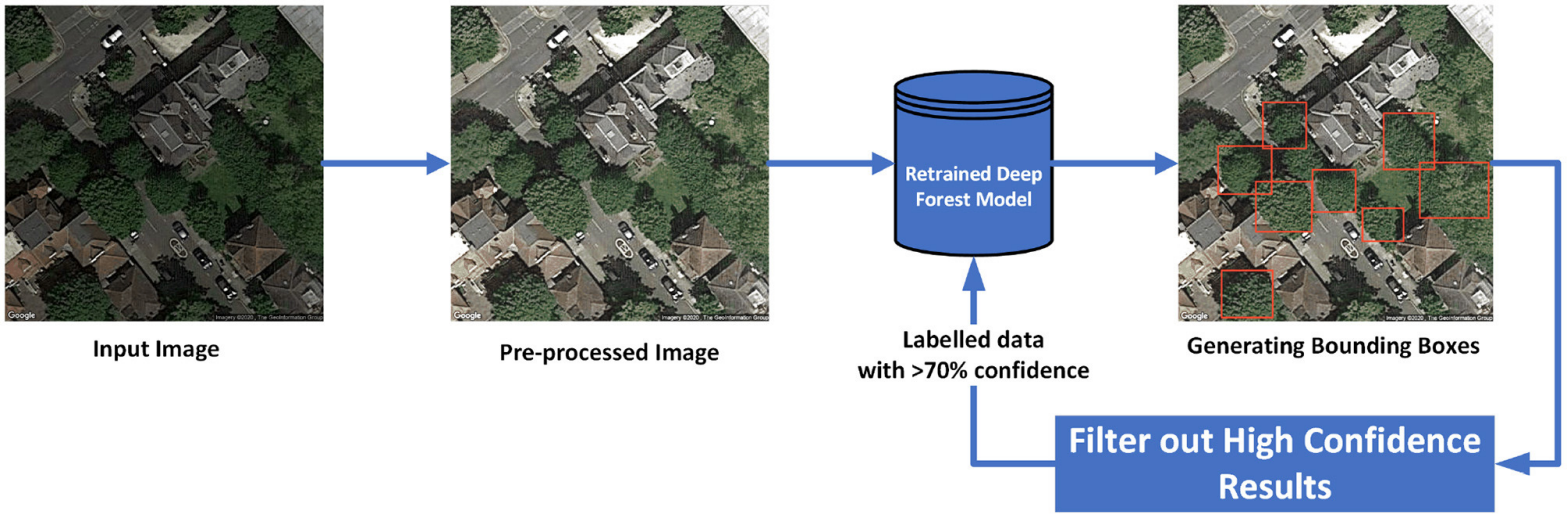
Tree recognition framework: self supervised training approach.

The proposed approach is illustrated in Figure 2. The training dataset were images from Camden (Borough in London, UK) and the unseen test dataset were images from a different city in the UK, Cambridge. The 150 test set images were hand labeled only to estimate the performance of the model. The dataset was initially normalized and enhanced using the pre-processing technique discussed in section 3. The normalization pipeline was followed by estimation of the tree crowns using the DeepForest models. It was seen that the performance was very poor as shown in Table 3. But even in these results, there were images that had very high confidence scores. These high confidence results (greater than a threshold of 70%) were filtered out from the training data. This generated a new set of around 1,500 automatically labeled training images which could be used to tune the DeepForest model. The backbone convolutional layers of the DeepForest model were frozen (as they act as feature extractors) and only the final fully connected layers are retrained with this data. The final results as seen in Table 3 shows considerable improvement in performance. The mean average precision (mAP) increased from 0.28mAP using un-tuned model to 0.89mAP with the retrained model for the intersection over union (IoU) threshold of 0.5. It should be noted that the hand corrected labels by Weinstein et al. (2019) could only achieve 0.61 for a similar setup and parameters. This is also presented in the Table 3 as a reference baseline. The proposed approach in this work did not use any hand labeling unlike the approach presented in Weinstein et al. (2019). Hence, could be deemed as successful method for recognizing tree crowns on any new dataset.

**Table 3:** Tree crown recognition results on images from Cambridge city.

**Type of model**	**Mean average performance**
Untrained DeepForest	0.28mAP
Proposed model (Self-supervised model)	0.89mAP
Weinstein et al. (2019) Baseline (Hand corrected labels)	0.61mAP

**Figure 3:**
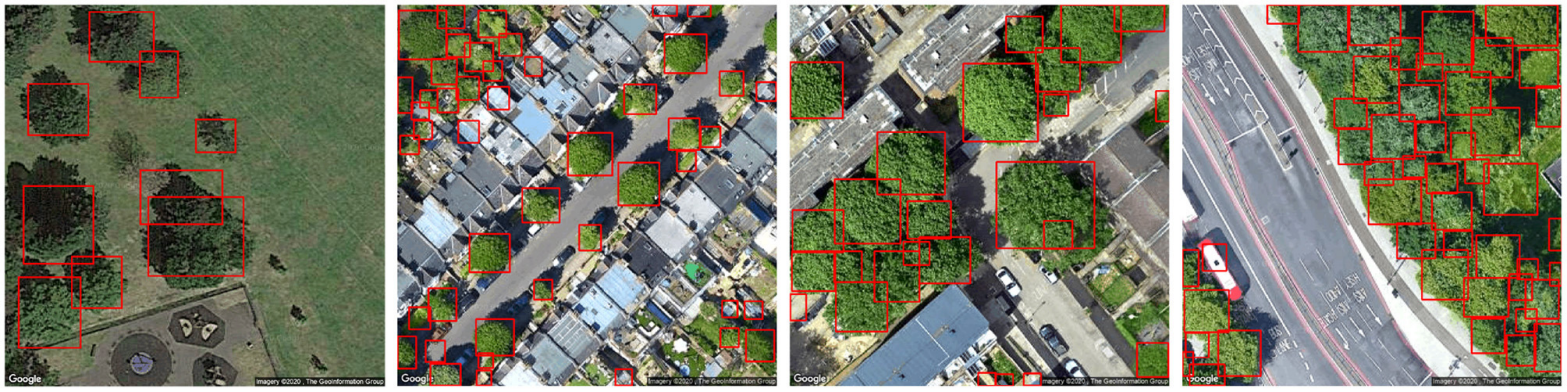
Positive results with self supervised model (trees in red bounding boxes).

**Figure 4:**
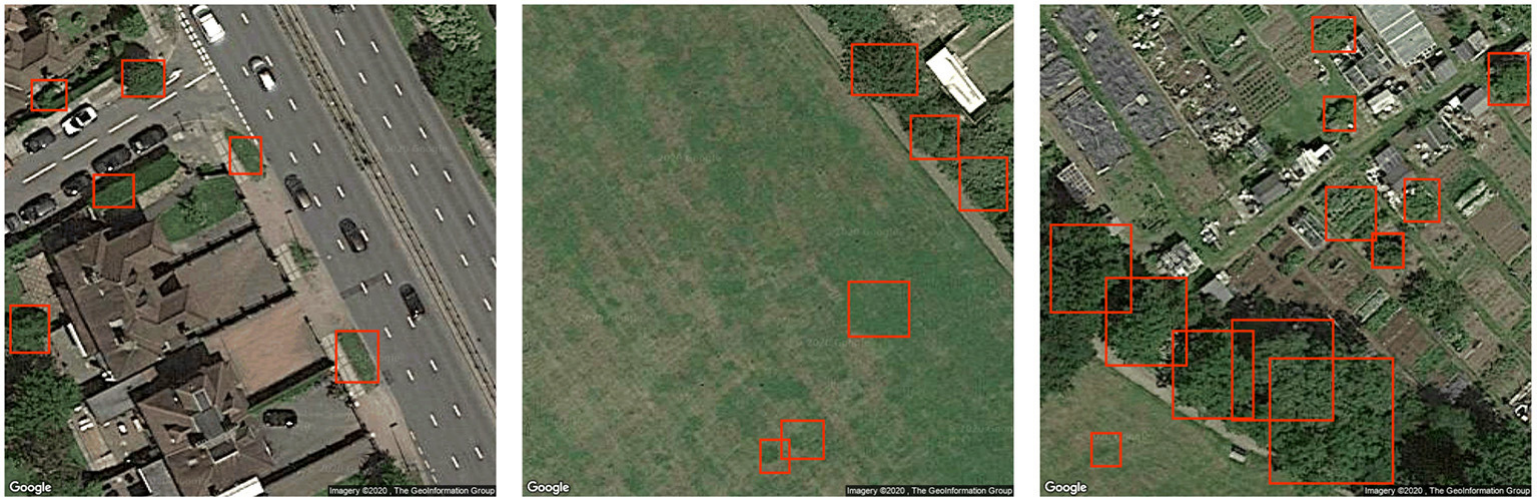
Some examples of undesirable results with self supervised model (trees in red bounding boxes).

Finally, qualitative analysis of tree recognition shows that the detection has considerably improved with the image pre-processing (normalization) as shown in Figure 1. The outputs on the self-supervised model are also analyzed, and the positive results recognizes most of the trees in the data even when they are sparsely or thickly populated as seen in Figure 3. Some very extreme cases of negative results are shown in Figure 4. It was hard to locate these negative examples from the test sets. Very few missed detection can be observed on some images. Tree cones were blurred in these cases which is the main distinguishing feature extracted by the DeepForest model. False positives may be triggered very rarely in special image lighting cases when a small round patch of grass resembles a tree cone as seen in the figure. But, it should be noted that in general the model does not trigger on grass and lawns as shown in Figure 3 and could effectively distinguish tree crowns from bushes and grass which is very impressive. The performance was acceptable for the proposed framework and the tree counts were estimated on the aerial view images at different radii around the pollutant monitoring stations to be incorporated into the framework.

## 5. Methodology

The standard methodology for air quality modeling is very similar to any other modeling task. The steps include collecting features from different data sources, pre-processing the data, analyzing the data, modeling the time series using different modeling techniques, and finally evaluating the model for performance. The different features are collected through the framework presented in Section 5.1 which can be scaled to any new city. The pre-processing and data analysis steps are summarized in Section 5.2 and finally modeling and prediction results are presented in the Section 5.4 followed by discussion of results in Section 6.

### 5.1. Global Framework Solution

**Figure 5:**
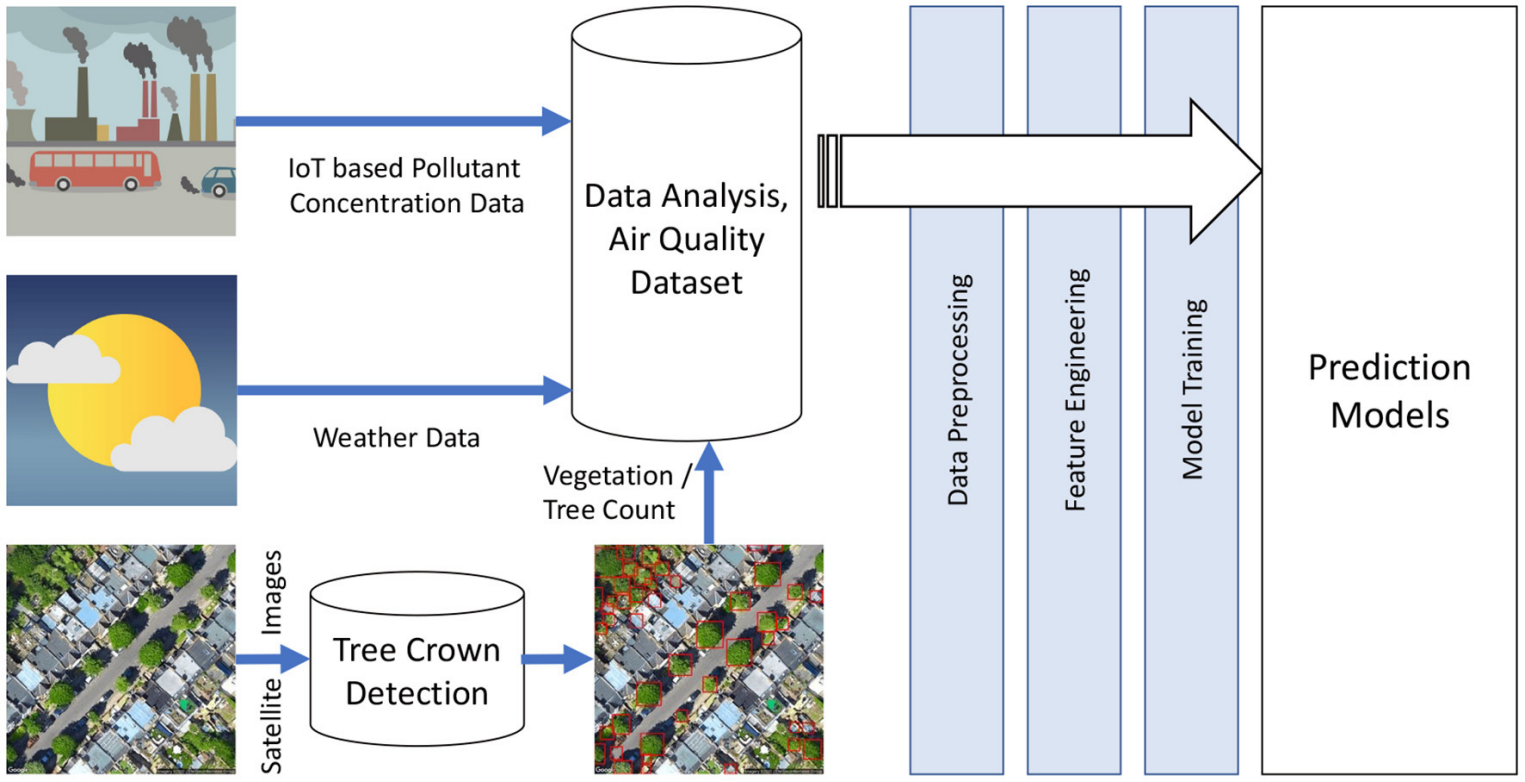
AirQuality framework: modeling pollutant concentration with weather and vegetation.

The framework developed for this work is shown in Figure 5. As explained earlier, it is easier to collect the pollutant concentration data in a developed urban city where government and other public organizations collect and publish this data. There is also a possibility of building cheaper air quality sensors as part of future improvements of this framework which is the next step of this research. The weather information is easily available from meteorological stations, and the vegetation information is collected based on the proposed tree crown detection presented earlier. This technique of vegetation detection can work for any new urban city as Google Earth images have been expanding to almost all international cities around the globe. There could be other factors like emissions that could be incorporated into the framework for future improvements.

### 5.2. Data Analysis

**Figure 6:**
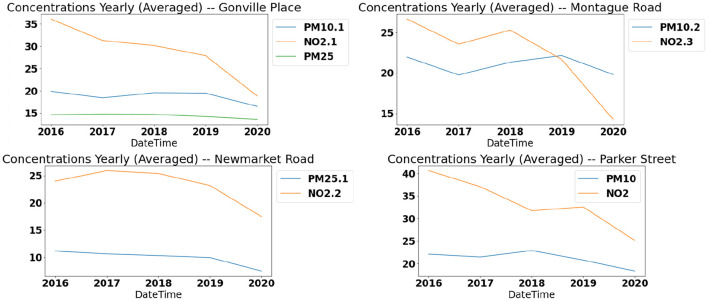
Yearly trends: pollutant concentration trends in the recent years.

From the initial analysis of the data sets, few interesting facts can be deduced. A declining trend is observed for the yearly mean concentration of both the pollutants. Figure 6 depicts the yearly concentrations for *NO*_2_ and *PM*_10_ at four locations in Cambridge between 2016 and 2020. The trend shows a steady decline in the pollutant concentration in Cambridge. The reduced concentrations in the year 2020 can be attributed to the restricted movements during lock-down due to the COVID-19 pandemic. Even without considering the influence of COVID-19 restrictions, the numbers have been reducing in general and with the lock-down and limited travel, the overall numbers had a very steep decline from the previous years.

**Figure 7:**
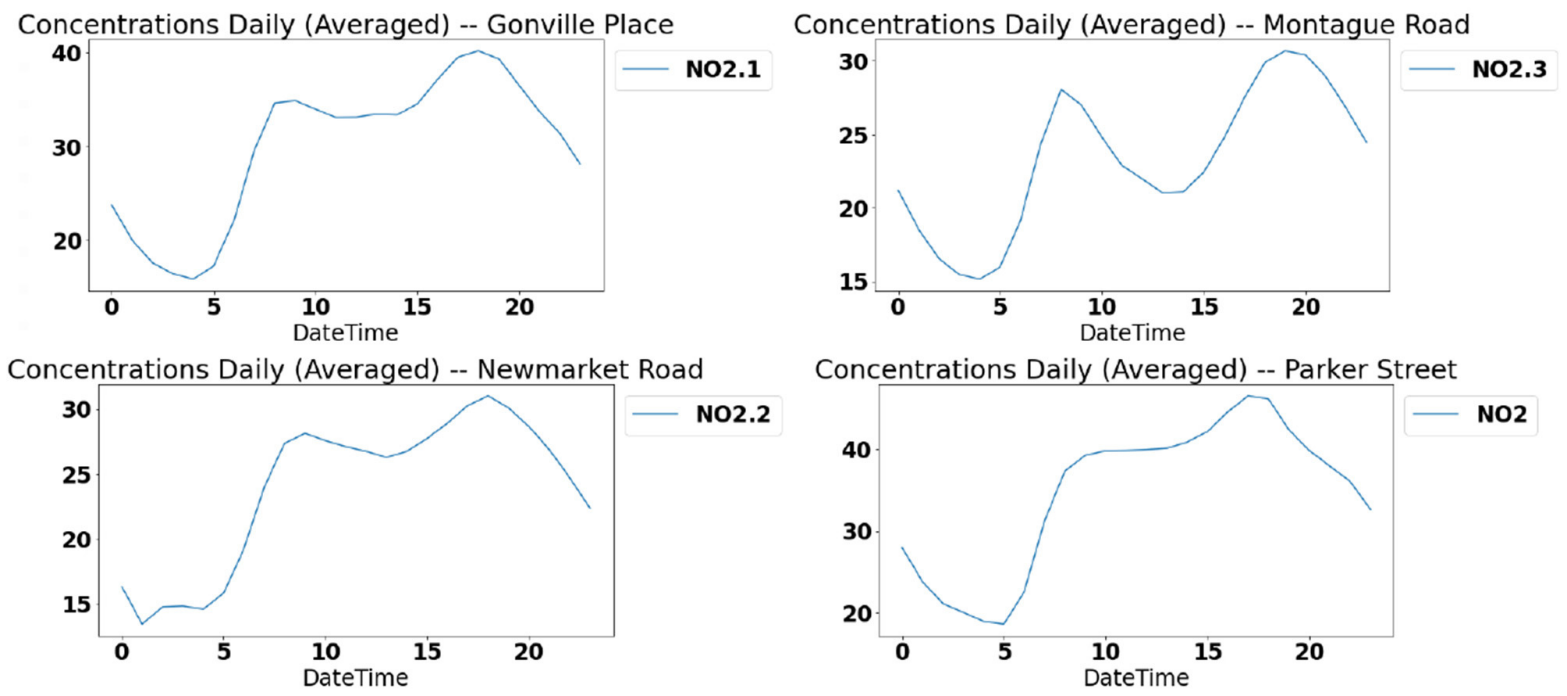
Daily *NO*_2_ trends: concentration trends across four different locations.

The daily distribution of *NO*_2_ across four different locations is shown in Figure 7. The common trend observed in all these figures is that the concentration reaches its peak around 18:00 which aligns with the peak traffic hour and the lowest concentration is observed in the early morning hours of 04:00 a.m. to 05:00 a.m. The concentration values show a steady increase throughout the day at all locations to reach their peak. From the peak at 18:00, it shows a trend of steady decline throughout the night to reach its lowest at early morning hours. This pattern can be attributed to the traffic pattern. Emission from traffic and other combustion are considered as the main sources of *NO*_2_. Figure 7 shows the strong correlation between traffic and *NO*_2_ concentration.

**Figure 8:**
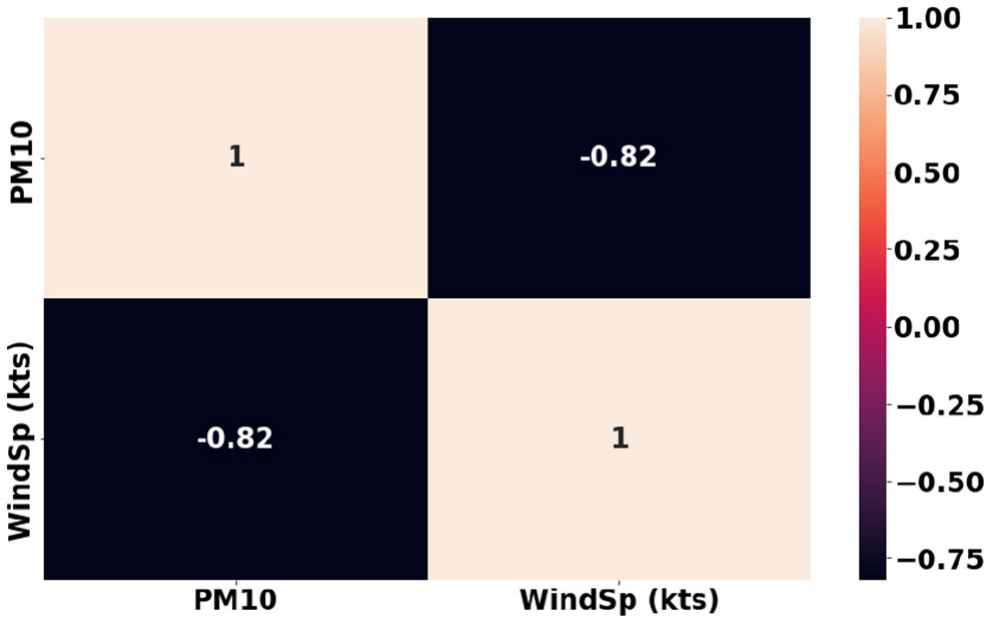
Correlation between Wind Speed and *PM*_10_.

**Figure 9:**
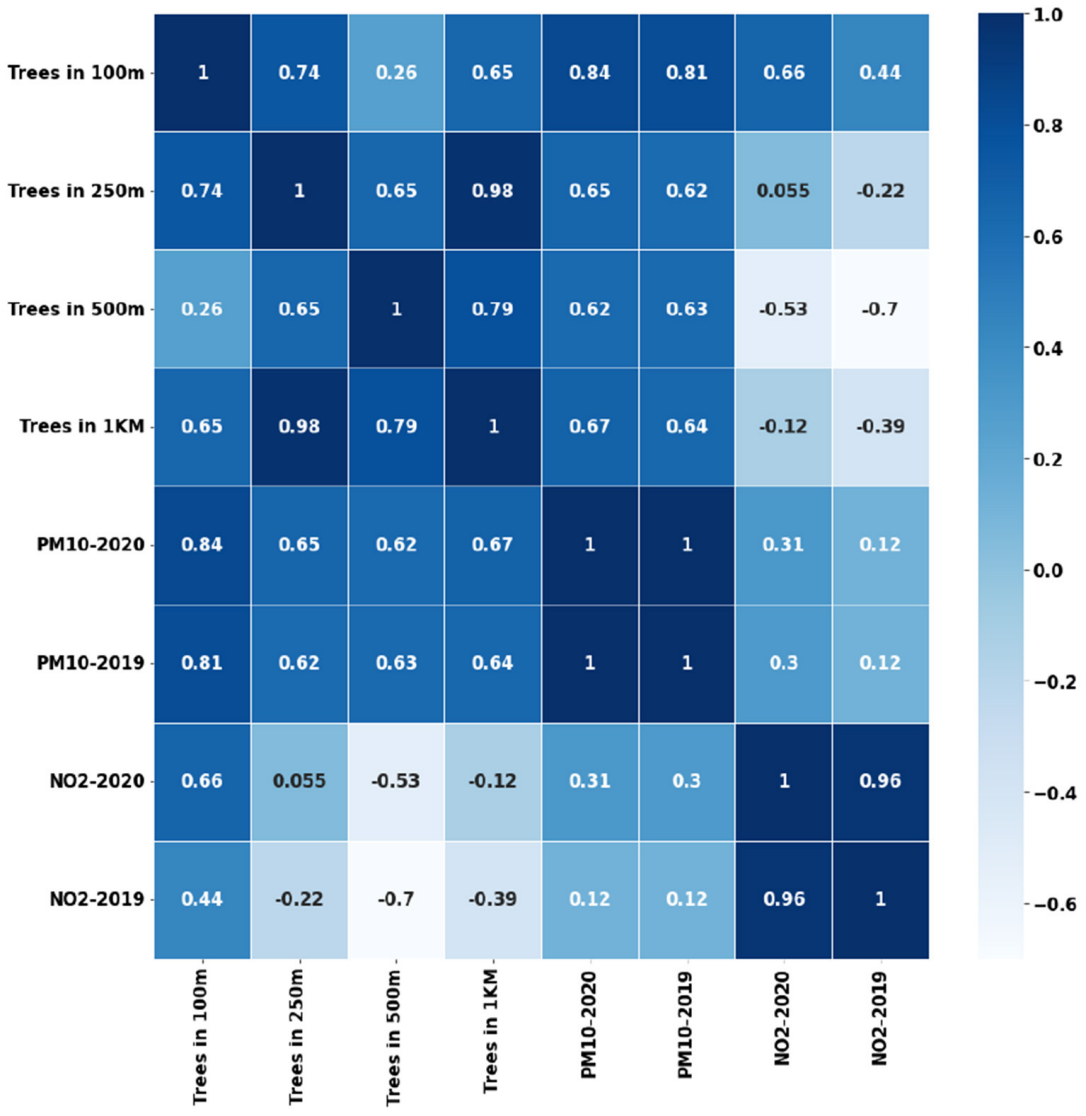
Correlation between vegetation and pollutants (*PM*_10_ and *NO*_2_).

Another interesting observation is the strong negative relation between Wind Speed and *PM*_10_ as shown in Figure 8. This has also been supported by many studies that the increase in wind speed blows away the particulate matter. Our earlier studies (Babu Saheer et al., 2020) using the data from London city have shown strong correlations between the vegetation and pollutants. A similar analysis using the tree counts around the pollutant monitoring stations in the city of Cambridge shows a good correlation with the pollutants under consideration as shown in Figure 9. The figure shows a yearly average of both gaseous pollutants, *NO*_2_ and particulate matter *PM*_10_, separately for the years 2019 and 2020. The tree counts are calculated at different radii around the pollutant monitoring station at the distances of 100 m, 250 m, 500 m, and 1 km. It can be seen from Figure 9 that the trees within a 100 m radius has strong correlations with both pollutants especially particulate matter.

### 5.3. Feature Engineering

**Figure 10:**
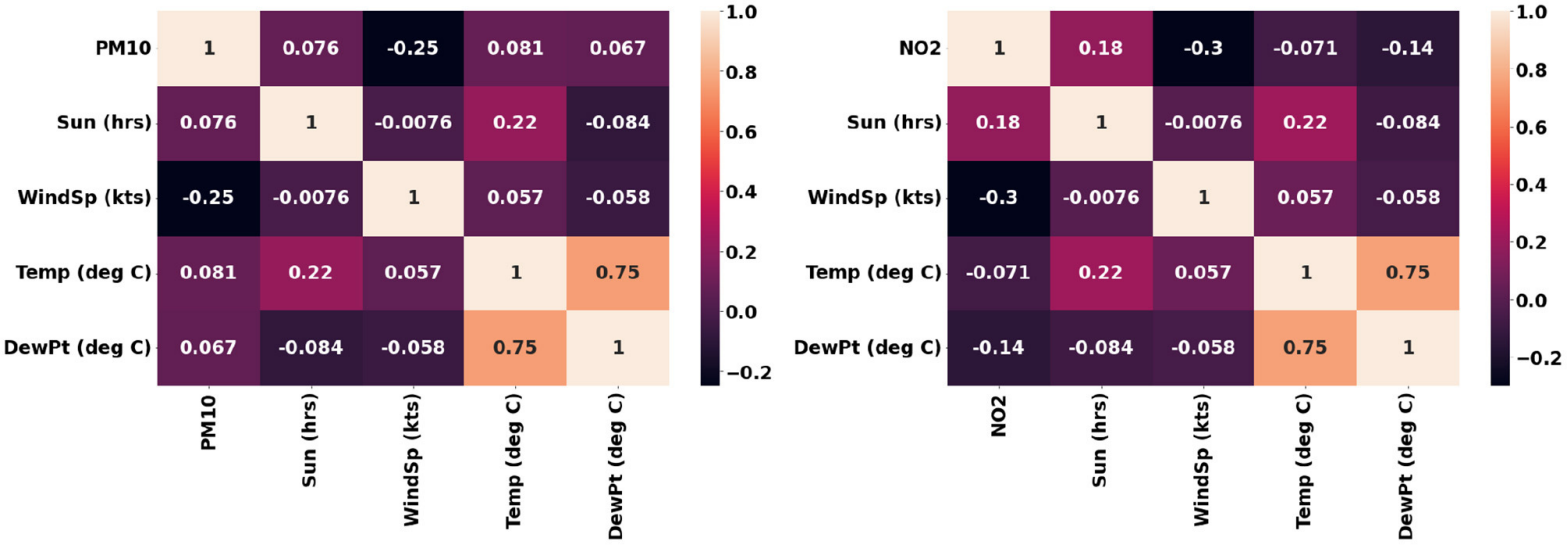
Correlation of the engineered features with regards to *PM*_10_ and *NO*_2_.

The air quality and weather data acquired from the Cambridge City Council was collated to generate a time series data of hourly intervals. These data points were used for modeling the gaseous and particulate matter pollutants based on the meteorological conditions and vegetation information in terms of the number of trees. Several new features were deduced from the existing ones as shown in Table 4. The date-time component in the data set was used to create new features like the day of the week, month number, and hour of the day. A Boolean variable indicating weekend or not was added to the data set. The mean pollutant concentration on weekends is substantially less than on weekdays. The 10 and 20 day rolling mean values for pollutant concentration was also added to the data set. A categorical variable denoting the 4 seasons were created and added to the data set. The month, hour, and weekday being cyclical variables, six trigonometric variables were created for these. The correlation of these new features with regards to the gaseous and particulate matter pollutant is presented in Figure 10. It can be observed that these features should ideally provide independent complimentary value to the models.

**Table 4:** Features introduced as a part of feature engineering.

**Feature**	**Data type**	**Description**
Weekend	Float (0/1)	Indicates whether the date is weekend or not
Weekday	Float (0/1)	Indicates whether the date is weekday or not
Season	String	The name of season derived from the date
HourCos,HourSin	Float	Since hour is a cyclic variable converted it to trigonometric functions Cos and Sin
MonthCos,MonthSin	Float	Since month is a cyclic variable converted it to trigonometric functions Cos and Sin
NO2MA10	Float	10 day Moving average of the concentration
NO2MA20	Float	20 Day Moving average of the concentration
100mTrees	Float	Number of trees within 100 m of the sensor
250mTrees	Float	Number of trees within 200 m of the sensor
500mTrees	Float	Number of trees within 500 m of the sensor
1000mTrees	Float	Number of trees within 1,000 m of the sensor

As shown in the table, the vegetation information is derived as the number of trees within different radii surrounding the location where the pollutant concentration was measured. This includes the number of trees within the radius of 100 m, 250 m, 500 m, and 1 km calculated using the tree recognition model described in Section 4.

### 5.4. Modeling and Prediction

This section discusses the results of experiments performed for modeling the data as described through the framework presented earlier (Figure 5). Experiments are performed with combinations of engineered features as mentioned in Section 5.3. Within this feature list, it can be observed that there are features pertaining to vegetation which denotes the number of trees within different distances from the location. Experiments were performed to compare the effects of vegetation by modeling air quality with and without these specific features pertaining to the vegetation information. All other newly engineered features were included in the different models except for Auto Regressive Integrated Moving Average (ARIMA) which is a uni-variate time series model for the specific pollutant concentration.

The pollutant data were collected from multiple locations within Cambridge between 2016 and 2020 with a value recorded every hour of the day. The data were divided into training data (2016–2019) and unseen test data (2019–2020) resulting in 28,000 (74%) data points for training and 9,785 (26%) data points for testing. The training dataset is further divided into two (at a ratio 80–20%) to generate training and cross-validation sets for tuning the hyperparameters. This research looks at multiple time series prediction models including machine learning regression models. The same dataset division was used in all models presented in this research. The final results are estimated on the same aforementioned held out test data set for all models.

The models investigated in this research range from the statistical Auto Regressive Integrated Moving Average (ARIMA) which is heavily used in air quality modeling studies to linear models like simple linear regression with the ridge, lasso, elastic-net counterparts alongside the polynomial regression. Non-linear models including SVR with polynomial and Radial Basis Function (RBF) Kernels and a combination of both were also tested. Deep Learning models, such as LSTM, are also investigated. All the aforementioned machine learning models were subject to hyperparameter optimization.

Similar to the other machine learning models, LSTM model was trained using the engineered features mentioned in the Section 5.3. An important factor to consider while training deep neural networks is the hyperparameter tuning. Hyperparameter optimization for the LSTM network was conducted using BayesianOptimization tuner available in the Keras library. The hyperparameters tuned are summarized in Table 5. The parameters were the number of neurons, learning rate, the loss function and the rate used in the dropout layer, and option to use dropout or not. After conducting 3 trials per set of parameters, the best set of hyperparameters was used to produce the results presented in the results table. The optimization resulted in Mean Squared Error as the identified loss function. The final LSTM network had four layers including 5 LSTM layers followed by a dense layer as the output. A dropout layer was added between these layers to prevent over fitting. The rate of the dropout layer was found using the tuner to be 0.4. The optimum number of neurons was calculated as 288, 480, 384, 100, and 50 for the 5 layers in the model and the best learning rate during the trials was 0.0001. Tanh activation function performed better than the others for the LSTM layers and linear activation function for the dense output layer. The LSTM model optimization is an ongoing task that demands a long training time and could be further improved. The aim of the experiments is to find the optimal model that could represent all the features in the framework. It could be identified that some of the common regression models might perform better than deep learning models like LSTM.

**Table 5:** Long short term memory (LSTM) Hyperparameters optimized during model training.

**Parameter name**	**Parameter values**
Number of LSTM layers	2 to 5
Number of Neurons per layer	32 to 512 with stepsize 32
Learning rate	1e-2, 1e-3 and 1e-4
Rate for dropout	between 0 and 0.5
Dropout option	True or False
Loss functions	MSE, MAE
Activation functions	Tanh, Linear, Relu, Sigmoid

Experiments are performed on gaseous pollutant (*NO*_2_) and particulate matter (*PM*_10_) separately. The models are generated for each location separately. As mentioned earlier, the air pollutant data is available with a frequency of every hour as average values for 5 years (from 2016 to 2020). But, there is only a single sample data value available for vegetation information per location in this entire time period. More data points are needed for modeling and understanding the influence of vegetation. Unfortunately, the air quality is monitored only at four locations. Initial experiments performed by combining two locations for training and the resulting model tested on a third location did not show promising results. As there are only data from four locations available currently, combining multiple locations and building more generic models is left as a future job. This paper presents results only for a couple of individual locations in Cambridge.

## 6. Results and Discussion

The results are presented as tables of error metrics. Five different error metrics are used to compare the performance of the models. These error metrics include mean average error(MAE), mean squared error (MSE), root mean squared error (RMSE), mean absolute percentage error (MAPE), and R2 score. While the errors (MSE, MAPE, RMSE, and MAE) are better lower, the R2-score ideally better closer to 1.

As mentioned earlier, readings from 2016 to 2019 are used as training data and the values from 2019 to mid-2020 are used as the test data. Tables 6, 7 show results for the different models of the gaseous pollutant *NO*_2_ for two individual locations. Tables 8, 9 present the same for the particulate matter *PM*_10_ for the same locations. The overall best performing models are highlighted in the tables. ARIMA models in all cases are uni-variate and use only concentration values and no other features (including engineered feature or vegetation information) for modeling. The other models presented in the tables including the LSTM models uses engineered features. These models are tested with and without the vegetation information (as explained earlier) represented by “With Trees” and “Without Trees” in the tables.

**Table 6:** Experimental results for NO2, location-1, and Parker Street.

**Model**	**Description**	**MAE**	**MSE**	**RMSE**	**R2**	**MAPE**
ARIMA	Without trees or extra features	**5.4968**	64.2431	8.0151	0.7804	29.2145
Linear regression	With trees	7.1151	86.9743	9.3260	0.6911	39.0577
Linear regression	Without trees	5.7071	59.4619	7.7111	0.7888	28.6116
LinearSVR	With trees	7.0114	85.4898	9.2460	0.6963	37.7552
LinearSVR	Without trees	5.7071	59.4619	7.7111	0.7888	28.6116
Polynomial regression	With Trees	5.7845	**59.2095**	7.6947	**0.7897**	30.7340
Polynomial regression	Without trees	5.7071	**59.4619**	7.7111	**0.7888**	28.6116
**Polynomial SVR**	**With Trees**	**5.7175**	59.6007	**7.7201**	0.7883	**28.9278**
**Polynomial SVR**	**Without trees**	**5.7071**	59.4619	**7.7111**	0.7888	**28.6116**
SVR With RBF Kernel	With trees	5.8804	62.1121	7.8811	0.7794	32.5764
SVR With RBF Kernel	Without trees	5.7071	59.4619	7.7111	0.7888	28.6116
PF-SVR with RBF Kernel	With trees	5.8081	61.4881	7.8414	0.7816	31.7735
PF-SVR with RBF Kernel	Without trees	5.7071	59.4619	7.7111	0.7888	28.6116
LSTM	With trees	6.8184	87.6507	9.3621	0.6822	30.6485
LSTM	Without trees	6.2056	71.3269	8.4455	0.7414	28.3280

*linearSVR, SVR with linear kernel; polynomial SVR, SVR with polynomial kernel; PF-SVR, SVR using polynomial features; SVR with RBF kernel, SVR with radial basis function (RBF) kernel; PF-SVR with RBF kernel, SVR using polynomial features and RBF kernel; LSTM, long short term memory; MAE, mean absolute error; MSE, mean squared error; RMSE, root MSE; R2, R-squared error; MAPE, mean absolute percentage error; SVR, support vector regression. Bold values are indicate the overall best performing models*.

**Table 7:** Experimental results for NO2, location-2, Gonville Place.

**Model**	**Description**	**MAE**	**MSE**	**RMSE**	**R2**	**MAPE**
ARIMA	Without trees or extra features	4.9797	51.3185	7.1636	0.7516	31.9924
Linear regression	With trees	6.8627	77.8650	8.8241	0.6172	40.6186
Linear regression	Without trees	6.8627	77.8650	8.8241	0.6172	40.6186
LinearSVR	With trees	6.5645	73.6806	8.5837	0.6378	37.8003
LinearSVR	Without trees	6.5697	73.7699	8.5889	0.6373	37.8582
Polynomial regression	With trees	5.8845	57.6263	7.5912	0.7167	34.9102
Polynomial regression	Without trees	5.7924	56.6969	7.5297	0.7213	33.9497
**Polynomial SVR Regression**	With Trees	**5.6715**	**56.1565**	7.4938	**0.7239**	**32.4848**
Polynomial SVR regression	Without trees	5.6841	56.3862	7.5091	0.7228	32.6777
SVR With RBF Kernel	With trees	5.8322	56.9608	7.5472	0.7200	36.4192
SVR With RBF Kernel	Without trees	5.8322	56.9608	7.5472	0.7200	36.4192
PF-SVR with RBF Kernel	With trees	5.7556	**55.6738**	**7.4615**	0.7263	36.0017
PF-SVR with RBF Kernel	Without trees	5.7361	55.7099	7.4639	0.7261	35.6012
LSTM	Without trees	5.0542	51.1251	7.1501	0.7484	23.0866
LSTM	With trees	5.2241	52.6281	7.2545	0.7410	25.0298

*LinearSVR, SVR with linear kernel; polynomial SVR, SVR with polynomial kernel; PF-SVR, SVR using polynomial features; SVR with RBF kernel, SVR with radial basis function (RBF) kernel; PF-SVR with RBF kernel, SVR using polynomial features and RBF kernel; LSTM, long short term memory; MAE, mean absolute error; MSE, mean squared error; RMSE, root MSE; R2, R-squared error; MAPE, mean absolute percentage error; SVR, support vector regression. Bold values are indicate the overall best performing models*.

**Table 8:** Experimental results for PM10, location-1, and Parker Street.

**Model**	**Description**	**MAE**	**MSE**	**RMSE**	**R2**	**MAPE**
ARIMA	Without Trees or Extra Features	3.8532	40.9184	6.3967	0.6375	59.7081
Linear Regression	With Trees	4.6605	40.9548	6.3996	0.4913	29.2084
Linear Regression	Without Trees	4.6605	40.9548	6.3996	0.4913	29.2084
LinearSVR	With Trees	4.4460	39.5361	6.2878	0.5089	27.0797
LinearSVR	Without Trees	4.4407	39.5027	6.2851	0.5093	27.0429
Polynomial Regression	With Trees	4.0152	32.2899	5.6824	0.5989	25.1360
Polynomial Regression	Without Trees	4.1887	34.1941	5.8476	0.5752	26.5016
Polynomial SVR Regression	With Trees	4.0677	33.2232	5.7640	0.5873	25.1261
Polynomial SVR Regression	Without Trees	4.0601	33.1445	5.7571	0.5883	25.0372
SVR With RBF Kernel	With Trees	3.9584	**31.9661**	**5.6539**	**0.6029**	**25.0983**
SVR With RBF Kernel	Without Trees	3.9584	**31.9661**	**5.6539**	**0.6029**	**25.0983**
PF-SVR with RBF Kernel	With Trees	**3.9552**	32.2535	5.6792	0.5993	25.0009
PF-SVR with RBF Kernel	Without Trees	3.9689	32.6011	5.7097	0.5950	25.0535
LSTM	With Trees	6.9262	79.6629	8.9254	0.0852	46.8802
LSTM	Without Trees	7.3973	94.0812	9.6995	0.0803	49.0300

*LinearSVR, SVR with linear kernel; polynomial SVR, SVR with polynomial kernel; PF-SVR, SVR using polynomial features; SVR with RBF kernel, SVR with radial basis function (RBF) Kernel; PF-SVR with RBF kernel, SVR using polynomial features and RBF kernel; LSTM, long short term memory; MAE, mean absolute error; MSE, mean squared error; RMSE, root MSE; R2, R-squared error; MAPE, mean absolute percentage error, SVR, support vector regression. Bold values are indicate the overall best performing models*.

**Table 9:** Experimental results for PM10, location-2, Gonville Place.

**Model**	**Description**	**MAE**	**MSE**	**RMSE**	**R2**	**MAPE**
ARIMA	Without Trees or Extra Features	3.5494	30.2370	5.4988	0.6762	23.1247
Linear Regression	With Trees	4.1726	31.8227	5.6411	0.5735	27.7313
Linear Regression	Without Trees	4.1711	31.8322	5.6420	0.5733	27.7040
LinearSVR	With Trees	4.1327	32.3228	5.6853	0.5668	26.8300
LinearSVR	Without Trees	4.1337	32.3442	5.6871	0.5665	26.8376
Polynomial Regression	With Trees	3.7524	**26.2176**	**5.1203**	0.6486	24.7285
Polynomial Regression	Without Trees	3.7730	26.5354	5.1512	0.6443	24.8110
Polynomial SVR Regression	With Trees	3.7695	26.7237	5.1695	0.6418	24.3154
Polynomial SVR Regression	Without Trees	3.7736	26.8158	5.1783	0.6406	24.4013
SVR With RBF Kernel	With Trees	**3.7202**	26.5390	5.1516	**0.6445**	24.2396
SVR With RBF Kernel	Without Trees	**3.7187**	26.5239	5.1501	**0.6445**	**24.0750**
PF-SVR with RBF Kernel	With Trees	3.7497	27.2717	5.2222	0.6345	24.1598
PF-SVR with RBF Kernel	Without Trees	3.7679	27.5793	5.2516	0.6303	24.2984
LSTM	With Trees	4.9231	45.8927	6.7744	0.4292	NA
LSTM	Without Trees	4.7463	44.2022	6.6484	0.4503	NA

*LinearSVR, SVR with linear kernel; Polynomial SVR, SVR with polynomial Kernel; PF-SVR, SVR using Polynomial features; SVR with RBF kernel, SVR with radial basis function (RBF) kernel; PF-SVR with RBF kernel, SVR using polynomial features and RBF kernel; LSTM, long short term memory; MAE, mean absolute error; MSE, mean squared error; RMSE, root MSE; R2, R-squared error; MAPE, mean absolute percentage error; SVR, support vector regression. Bold values are indicate the overall best performing models*.

As observed from Tables 6, 7, SVR models with polynomial Kernel performs slightly better than the other counterparts for the *NO*_2_ modeling. The influence of trees on this pollutant was especially noticed in one of the two locations. But more experiments with data points combined from multiple locations need to be performed to understand the influence of vegetation. The tree feature might be acting just as a prior. The *PM*_10_ models in Tables 8, 9 show similar trends for Support Vector Regression with the RBF Kernel. Again the effects of vegetation is being noticed for one of the locations on some error metrics.

The ARIMA models in most cases is showing slightly better performance on one or two error metrics, but ARIMA is limited by the fact that it looks at only the time series trend of the pollutant value alone and more features cannot be included in this uni variate model. Deep learning models like LSTM at this point do not show a significant performance improvement, but has the potential to be tuned further with more data, more features and better parameter and hyperparameter optimization.

## 7. Conclusion and Future Work

This research proposes a novel framework for the air quality modeling considering the related factors of weather and vegetation. The prototype framework was validated for the city of Cambridge using the existing pollutant data monitored by the city authorities and common weather measurements. Models were tested for two different locations within the city. The vegetation information was incorporated into the framework with our own novel methodology of self-supervised tree detection system based on Google Earth Satellite images. Multiple Machine Learning systems were modeled for a gaseous and a particulate matter pollutant. Models ranged from statistical ARIMA models to various linear and non-linear regression techniques including SVR with different Kernels and an advanced LSTM based deep learning model. Multiple error metrics were analyzed to understand the overall performance of the model. The SVR models show promising results even with the lack of localized weather conditions and lack of data from multiple locations for effective use of the vegetation feature. The deep learning models also show some prospects for improvement with more appropriate data and optimization.

Our current research is focused on building custom pollutant monitoring devices to collect data from multiple locations within the city to generate more accurate and generic models. We aim to look at local weather conditions and the effects of micro climate on the model. The research will also be expanded to other types of pollutants to understand various features affecting the pollutant concentrations. Estimates of emissions may also be incorporated into the framework along with tree species or vegetation or terrain type information. With more data collected, the research will focus on improving models including the deep learning models. The framework can also be scaled to any other city in the world. Different seasonal variations (currently only incorporated as a single feature value as season) will also be studied. The tree species identification from aerial view images has already been initiated (Waters et al., 2021) and would also be incorporated in the framework. Micro climate modeling using custom monitoring devices measuring local weather conditions and more pollutants are also pursued as future steps in this research. There are plans to acquire more aerial view data using drone imagery to model the variations in seasonality of the vegetation. The research is underway along these lines with an aim to continuously improve this framework.

## Data Availability Statement

Publicly available datasets were analyzed in this study. This data can be found here: https://www.airqualityengland.co.uk/local-authority/?la_id=51; https://developers.google.com/earth-engine/guides/exporting; https://www.cl.cam.ac.uk/research/dtg/weather/index-daily-text.html.

## Author Contributions

LB contributed to the conception, design and implementation of the study, and wrote the first draft of the manuscript. MM contributed to the literature review. AB contributed to the experiments and derived results for the study. JZ contributed to the data analysis and preparation of the study. AB, MM, and JZ wrote sections of the manuscript. All authors contributed to manuscript revision, read, and approved the submitted version.

## Conflict of Interest

The authors declare that the research was conducted in the absence of any commercial or financial relationships that could be construed as a potential conflict of interest.

## Publisher's Note

All claims expressed in this article are solely those of the authors and do not necessarily represent those of their affiliated organizations, or those of the publisher, the editors and the reviewers. Any product that may be evaluated in this article, or claim that may be made by its manufacturer, is not guaranteed or endorsed by the publisher.
